# Longitudinal validation of the PROMIS-16 in a sample of adults in the United States with back pain

**DOI:** 10.1007/s11136-024-03826-6

**Published:** 2024-11-06

**Authors:** Anthony Rodriguez, Chengbo Zeng, Ron D. Hays, Patricia M. Herman, Maria O. Edelen

**Affiliations:** 1 RAND Corporation, Behavioral and Policy Sciences, 20 Park Plaza #910, Boston, MA USA; 2 Patient Reported Outcomes, Value and Experience (PROVE) Center, Department of Surgery, Brigham and Women’s Hospital, Boston, MA USA; 3 Division of General Internal Medicine and Health Services Research, UCLA Department of Medicine, Los Angeles, CA USA; 4 RAND Corporation, Behavioral and Policy Sciences, 1776 Main Street, Santa Monica, CA USA

**Keywords:** PROMIS-16, PROMIS-29 + 2, Patient-reported outcomes, Short form validation, Health-related quality of life

## Abstract

**Purpose:**

This longitudinal study evaluates whether the Patient-Reported Outcomes Measurement and Information System (PROMIS)-16 domains capture average change over time comparable to the PROMIS-29 + 2 and have similar associations with change in overall health rating and two disability indices.

**Methods:**

Data were collected using Amazon’s Mechanical Turk at baseline, 3 months, and 6 months among individuals reporting chronic low back pain. The analytic sample includes respondents who completed baseline and at least one follow-up assessment (*N* = 1137). We estimated latent growth models for eight PROMIS domains and compared growth parameters between the PROMIS-16 and PROMIS 29 + 2 with a z-test. Additionally, for each domain, random intercept and slope scores for individuals were computed for the PROMIS-29 + 2 and PROMIS-16 and correlated to estimate concordance. Using growth parameters for physical function and pain interference, we predicted average change in the Oswestry Disability Index (ODI), Roland Morris Disability Questionnaire (RMDQ), the overall health rating, and compared regression coefficients between the PROMIS-16 and PROMIS 29 + 2.

**Results:**

All growth models fit the data well. Intercept and slope parameters were statistically comparable (p’s > 0.05) in magnitude across all domains between the PROMIS-16 and PROMIS-29 + 2. Correlations between random intercept and slope scores for individuals across domains were high. Additionally, the regression coefficients between slopes for pain interference and physical function and ODI, RMDQ, and overall health rating were statistically comparable (p’s > 0.05) between the PROMIS-16 and PROMIS 29 + 2.

**Conclusion:**

Results provide between-level support for the longitudinal and predictive validity of the PROMIS-16. Similar average baseline scores and changes over time were observed between PROMIS-16 and PROMIS-29 + 2. Further, average change estimates comparably predicted average change in distal outcomes. This work provides evidence supporting the utility of the PROMIS-16 as a viable, short-profile option for use in clinical and research settings.

**Supplementary Information:**

The online version contains supplementary material available at 10.1007/s11136-024-03826-6.

## Introduction

The development of the Patient-Reported Outcome Measurement Information System (PROMIS^®^) [1, 2] was supported by a National Institutes of Health initiative to evaluate and monitor physical, mental, and social health. PROMIS measures are constructed following established guidelines for patient-reported outcome measures (PROM) development and evaluation [3]. The PROMIS library includes a variety of measurement options, including numerous domain measures that can be administered individually as fixed-length short forms as well as pre-packaged forms called PROMIS Profiles that assess seven multi-item domains: anxiety, depression, fatigue, pain interference, physical function, sleep disturbance, and ability to participate in social roles and activities, and include a single item measure of pain intensity. The seven domains are assessed with 4, 6, or 8 items each, resulting in Profile measures of 29, 43, or 57 items [4]. Moreover, the domains can be combined into mental and physical health summary scores [5, 6]. The PROMIS Profiles have seen increased adoption in clinical and health research settings [4, 7] given their efficiency, flexibility, and sensitivity, as well as their ability to characterize specific health domains and generate summary scores [8].

Until recently, the shortest options were the PROMIS-29 or PROMIS-29 + 2 [2, 4], which adds two items for the cognitive function-abilities domain. However, concerns over the burden of overall length may lead some to opt for alternative shorter measures such as the Global-10 [9]. While shorter, the Global-10 lacks the domain score specificity of the longer PROMIS-29. In response, Edelen et al. [10] developed the PROMIS-16, an ultra-brief measure spanning the same eight health-related quality of life domains as the PROMIS 29 + 2, using only two items per domain. Given that this measure is minimally burdensome and can generate domain-specific scores, physical and mental summary scores, and PROMIS preference scores [6, 10], there is a utility for this measure in research and routine clinical care. Initial validation work by Zeng et al. [11] found that correlations among corresponding PROMIS-16 and the PROMIS-29 + 2 scores were strong and mean scores were similar. But, no studies have yet compared longitudinal change over time in PROMIS-16 and PROMIS 29 + 2 domain scores.

In clinical settings, repeated data collection allows for evaluating progress or decrements in each domain. Similarly, in research applications, tracking change as a function of an intervention is critical in determining whether the intervention is successful at changing a given outcome. The latent growth model (LGM) framework [12, 13] is ideal for examining longitudinal processes and changes over time. Briefly, LGMs are designed to use repeated measurements to estimate growth factors - an intercept, most commonly a baseline score, and, at a minimum, a slope that characterizes linear change over time. With these parameters, we can identify the average initial status and the direction and magnitude of the average change over time. These methods are regularly applied to PROMIS data in intervention and observational research [14, 15] and clinical settings [16, 17]. While shorter PROMs are appealing in clinical and research settings, the question remains whether the same observable effects or change can be recovered with the PROMIS-16 ultra-short profile.

Using a sample of respondents with back pain, this work aims to evaluate the validity of the newly developed PROMIS-16 by comparing its mean baseline scores and longitudinal change estimates to the commonly used PROMIS 29 + 2 Profile measure. We also assess the predictive validity (i.e., the ability of a test or measurement to predict a future outcome) of the PROMIS-16 by comparing the statistical significance and magnitude of effects between latent growth factors derived from the PROMIS-16 versus the PROMIS-29 + 2 and several outcome measures (i.e., overall health rating and two disability indices). Specifically, the focus of this work is to evaluate the comparability of between-level parameter estimates (i.e., average initial status and average change) from the PROMIS-16 and PROMIS 29 + 2 as well as between-level prediction – i.e., the average change of PROMIS domains predicting average change of outcomes.

## Method

### Data source and study sample

This study uses data from an online nonprobability convenience sample obtained from Amazon’s Mechanical Turk (MTurk) internet panel, collected in 2021 [18]. Briefly, we recruited participants who met the following criteria: (1) aged 18 years or older, (2) having a United States IP address, and (3) having completed at least 500 MTurk “human intelligence tasks” with a minimum approval rate of 95%. Participants who consented and enrolled in the baseline assessment were required to complete a general health questionnaire, which included demographic characteristics, clinical information, and PROMIS item-level data. Those reporting back pain were further asked to complete a back pain survey and were invited to participate in the 3- and 6-month follow-up surveys. Participants who only completed the general health questionnaire were paid $1.50; those who completed the back pain survey received an additional $2. To ensure data quality, we included two fake conditions (“Chekalism” and “Syndomitis”) [19]. A total of 6997 participants consented and enrolled in the assessment, of whom 247 were excluded because they did not complete the baseline survey, and 975 were excluded because they endorsed one or both fake conditions, resulting in 5775 in the baseline analytic sample. Among them, 2326 reported back pain and were offered the back pain survey, with 1972 completing the back pain portion. Of these, 54.6% completed the 3-month survey and 42.8% completed the 6-month survey. Individuals who did not complete the 3-month survey were still invited to participate in the 6-month follow-up. Inclusion in this analytic sample required that an individual complete a baseline and at least one follow-up assessment. Thus, the final analytic sample (*n* = 1137) includes individuals who reported having back pain and completed baseline and at least one follow-up assessment. Respondent retention from baseline to 3 months was not significantly associated with gender, age, race, income, and education. Retention from baseline to 6 months was also not associated with gender, race, income, and education; however, it was associated with age (OR = 1.032).

### Measures

#### PROMIS-29 + 2 profile

The PROMIS^®^-29 + 2 profile assesses seven health domains (i.e., physical function, fatigue, pain interference, depressive symptoms, anxiety, ability to participate in social roles and activities, and sleep disturbance) using four items per domain. It also contains two items to measure cognitive function – abilities. A single pain intensity item is also included but was not used in these analyses. We treat the PROMIS 29 + 2 as the gold standard for all analyses [4]. PROMIS domain scores were generated for all eight domains using established parameters from the PROMIS item banks (parameters for the sleep-related impairment item were generated based on calibration to the sleep disturbance items). All domains were centered with a mean of 50 and SD of 10 (i.e., T-score metric). Except for cognitive function-abilities and physical function, higher scores indicate poorer health. Additional details about PROMIS measures and scoring can be found at https://www.healthmeasures.net/explore-measurement-systems/promis.

#### PROMIS^®^-16 profile

The PROMIS^®^-16 is a recently developed measure evaluating eight health domains with two items each: physical function, fatigue, pain interference, depressive symptoms, anxiety, ability to participate in social roles and activities, sleep disturbance, and cognitive function-abilities [10]. Twelve of the 16 items are also in the PROMIS-29 + 2: both PROMIS-16’s items for physical function, fatigue, pain interference, depressive symptoms, and anxiety are included in the PROMIS-29 + 2; and one of the two items in the PROMIS-16 for ability to participate in social roles and activities, and cognitive function – abilities domains are from the PROMIS-29 + 2. Sleep disturbance does not include any items from the PROMIS-29 + 2. Domain scores were computed using the same procedures described previously.

#### The Oswestry Disability Index (ODI)

The ODI is a 10-item measure assessing pain intensity, personal care, lifting, walking, sitting, standing, sleeping, sex life (if applicable), social life, and traveling. Response options range from 0 to 5, with higher scores indicating more disability. The scale is scored by summing scores across all items, dividing the total score by the maximum possible, and multiplying by 100. The ODI score can also be classified into five severity groups [20].

#### Roland Morris Disability Questionnaire (RMDQ)

The RMDQ is a 24-item measure assessing the impact of back pain on 24 daily activities. The scale score can range from 0 (no disability) to 24 (maximum disability) [21].

#### Overall health rating

The general health item “In general, would you say your health is (Poor = 1 to Excellent = 5) was taken from the PROMIS^®^ Global Health [22].

### Analytic plan

Latent growth models for each of the eight domains were first estimated separately for PROMIS-16 and PROMIS-29 + 2 (8 domains * 2 profiles = 16 models total) and evaluated by model fit indices: chi-square, root mean error of approximation (RMSEA), comparative fit index (CFI), and standardized root mean residual (SRMR) to ensure adequate fit before subsequent analyses. CFI values of 0.95 or larger indicate good fit, and RMSEA values less than 0.09 and SRMR values less than 0.08 represent acceptable fit [23, 24]. For each domain, random intercept and slope scores for individuals were computed for the PROMIS-29 + 2 and PROMIS-16, saved, and then correlated to estimate the degree of concordance. Next, we evaluated the longitudinal comparability of each of the eight PROMIS-16 domain scores to the PROMIS-29 + 2. Specifically, PROMIS-16 and PROMIS-29 + 2 intercept and slope growth factors from each domain were statistically compared using z-tests [25, 26] and evaluated at *p* < .05. This process was repeated for each PROMIS domain. After evaluating all growth parameters (i.e., intercept and average change), we tested the longitudinal predictive validity of the growth parameters using two PROMIS domains (Physical Function and Pain Interference) which have been previously found to be associated with the RMDQ, ODI, and overall health rating [27–29]. Given that predictive validity is focused on evaluating a measurement or score predicting an outcome, we treat intercepts and slopes of PROMIS Physical Function and Pain Interference as predictors of average change in three longitudinal outcomes: RMDQ, ODI, and overall health rating. To determine whether PROMIS-16 and PROMIS-29 + 2 domains comparably predicted change in outcomes longitudinally, we estimated parallel process models where the slope of the PROMIS domain was used to predict the slope of the outcome measure (e.g., average change of Physical Function predicting average change of the ODI). Models were run separately for the PROMIS-16 and PROMIS-29 with each longitudinal outcome. Regression coefficients from each domain were statistically compared using z-tests [25, 26] and evaluated at *p* < .05. All models were estimated in Mplus v8.10 [30] using maximum likelihood estimation with robust standard errors, accommodating continuous and ordinal variables and missing data and providing unbiased and consistent estimates.

## Results

Sociodemographic descriptives are presented in Table 1 to characterize the sample. The sample was primarily White (85%), with 9% identifying as Hispanic, and consisted of slightly more females (54%) than males. Over half of the participants (55%) were educated with a bachelor’s degree or higher and had an annual income of $50,000 or more (51%). The median age was 40 years (Interquartile range: 33–51). A complete set of PROMIS-16 and PROMIS-29 + 2 domain means and standard deviations, along with longitudinal outcome descriptives, is presented in Table S1 in Supplemental Materials. We include spaghetti plots of individual trajectories for PROMIS-16 and PROMIS-29 + 2 domains, ODI, RMDQ, and overall health rating in Supplemental Materials.

**Table 1:** Demographic characteristics of the analytic sample (*N* = 1137)

Characteristics	*N* (%)
**Age (years**,** median**,** IQR)**	40 (33, 51)
**Race**	
White	966 (85)
Black or African American	101 (9)
Asian or Asian American	80 (7)
Native Hawaiian or Pacific Islander, Native American, Other races	28 (2)
Multiracial	37 (3)
**Ethnicity**	
Non-Hispanic	1031 (91)
Hispanic	106 (9)
**Gender**	
Female	611 (54)
Male	519 (46)
Transgender	2 (0)
Do not identify as female, male, or transgender	5 (0)
**Education**	
Bachelor’s degree or higher	625 (55)
**Annual income**	
Less than $49,999	560 (49)
$50,000 - $99,999	417 (37)
More than $100,000	160 (14)

Model fit indices for separate growth models for PROMIS-16 and PROMIS 29 + 2 by domain are presented in Table 2. All models fit the data well. The concordance of a random intercept and slope scores for individuals between PROMIS-16 and PROMIS-29 was computed. For random intercepts, correlations between PROMIS-16 and PROMIS-29 + 2 were as follows: Anxiety (*r* = .95), Cognitive Function (0.87), Depression (0.98), Fatigue (0.98), Pain Interference (0.98), Physical Function (0.94), Sleep Disturbance (0.89), and Social Roles (0.95). For random slopes, correlations between PROMIS-16 and PROMIS-29 + 2 were as follows: Anxiety (0.95), Cognitive Function (0.61), Depression (0.91), Fatigue (0.91), Pain Interference (0.94), Physical Function (0.73), Sleep Disturbance (0.69), and Social Roles (0.68).

**Table 2:** Model fit indices for latent growth models by domain for both PROMIS-16 (P16) and PROMIS-29 + 2 (P29 + 2)

	χ^2^	RMSEA	CFI	SRMR
**Anxiety**				
P16	10.746 (*p* = .013)	0.048	0.990	0.032
P29 + 2	7.613 (*p* = .055)	0.037	0.995	0.014
**Cognitive Function**				
P16	1.203 (*p* = .273)	0.013	1	0.008
P29 + 2	0.076 (*p* = .783)	0	1	0.002
**Depression**				
P16	1.248 (*p* = .264)	0.015	1	0.005
P29 + 2	3.499 (*p* = .061)	0.047	0.998	0.009
**Fatigue**				
P16	3.084 (*p* = .079)	0.043	0.998	0.010
P29 + 2	10.455 (*p* = .002)	0.047	0.993	0.056
**Pain Interference**				
P16	1.088 (*p* = .297)	0.009	1	0.005
P29 + 2	0.874 (*p* = .350)	0	1	0.004
**Physical Function**				
P16	2.638 (*p* = .104)	0.038	0.998	0.009
P29 + 2	15.455 (*p* = .002)	0.060	0.985	0.022
**Sleep disturbance**				
P16	0.455 (*p* = .500)	0	1	0.004
P29 + 2	0.081 (*p* = .775)	0	1	0.002
**Social Roles**				
P16	0.05 (*p* = .823)	0	1	0.002
P29 + 2	0.209 (*p* = .647)	0	1	0.003

Note: RMSEA: Root mean error of approximation, CFI: Comparative fit index, SRMR: Standardized root mean squared residual

### Longitudinal validity

Growth parameters from parallel process models using the PROMIS-16 and PROMIS 29 + 2 scores for each domain are presented in Table 3. There were significant differences in the intercept parameter between PROMIS-16 and PROMIS 29 + 2 across all domains, except for anxiety, depression, and pain interference. That said, the magnitude of differences was small. In the T-score metric (i.e., Mean = 50, SD = 10), the difference between intercepts ranged from 0.12 (depression) to 1.29 (physical function). When transformed to Cohen’s d effect sizes, this range was from 0.02 to 0.13, with all but two being < 0.10 – all falling below the 0.2 small effect threshold [31]. On the other hand, for the slopes (i.e., average change), there were no significant differences between PROMIS-16 and PROMIS 29 + 2 across all domains. For all but two domains, there was no significant average change across the three measurement time points—i.e., the slopes were not significantly different than zero. However, on average there was a significant decrease in physical function and pain interference over time.

**Table 3:** PROMIS-16 (P16) and PROMIS-29 + 2 (P29 + 2) growth parameters for all PROMIS domains and Wald tests of Equality constraints between profiles

	Intercept M (SE)	Slope M(SE)
**Anxiety**		
P16	56.161 (0.285), *p* < .001	0.166 (0.117), *p* = .156
P29 + 2	55.995 (0.281), *p* < .001	0.083 (0.111), *p* = .457
*z-score*,* p-value*	z = 0.41, *p* = .341	z = 0.51, *p* = .610
**Cognitive Function**		
P16	48.752 (0.236), *p* < .001	0.180 (0.121), *p* = .135
P29 + 2	49.511 (0.224), *p* < .001	0.136 (0.122), *p* = .267
*z-score*,* p-value*	z = -2.33, *p* = .019	z = 0.26, *p* = .795
**Depression**		
P16	55.054 (0.292), *p* < .001	-0.069 (0.117), *p* = .553
P29 + 2	54.936 (0.295), *p* < .001	-0.064 (0.114), *p* = .576
*z-score*,* p-value*	z = 0.28, *p* = .779	z = -0.03, *p* = .910
**Fatigue**		
P16	52.650 (0.273), *p* < .001	0.169 (0.116), *p* = .147
P29 + 2	53.673 (0.285), *p* < .001	0.166 (0.113), *p* = .144
*z-score*,* p-value*	z = -2.59, *p* = .010	z = 0.02, *p* = .976
**Pain Interference**		
P16	55.265 (0.234), *p* < .001	-0.249 (0.115), *p* = .030
P29 + 2	55.005 (0.241), *p* < .001	-0.222 (0.115), *p* = .053
*z-score*,* p-value*	z = 0.77, *p* = .441	z = -0.17, *p* = .865
**Physical Function**		
P16	48.271 (0.239), *p* < .001	-0.539 (0.097), *p* < .001
P29 + 2	46.986 (0.255), *p* < .001	-0.610 (0.094), *p* < .001
*z-score*,* p-value*	z = 3.68, *p* < .001	z = 0.53, *p* = .596
**Sleep disturbance**		
P16	52.250 (0.240), *p* < .001	0.150 (0.112), *p* = .180
P29 + 2	53.187 (0.262), *p* < .001	0.058 (0.114), *p* = .612
*z-score*,* p-value*	z = 2.64, *p* = .008	z = 0.58, *p* = .562
**Social Roles**		
P16	50.029 (0.263), *p* < .001	0.022 (0.116), *p* = .846
P29 + 2	50.994 (0.268), *p* < .001	-0.111 (0.113), *p* = .325
*z-score*,* p-value*	z = -2.57, *p* = .010	z = 0.82, *p* = .412

### Predictive validity

Table 4 presents regression coefficients for changes (i.e., slopes) in pain interference and physical function slopes predicting changes (i.e., slopes) in the ODI, RMDQ, and the overall health rating. Regression coefficients were not significantly different for all analyses between PROMIS-16 and PROMIS-29 + 2.

**Table 4:** Predictive validity regression coefficients for PROMIS-16 (P16) and PROMIS-29 + 2 (P29 + 2) slopes Predicting Outcome slopes

	Pain Interference		Physical Function	
Outcomes	P16	P29 + 2	P16	P29 + 2
***Oswestry Disability Index***				
	2.69 (0.61) *p* < .001	2.63 (0.58) *p* < .001	-2.39 (0.54) *p* < .001	-2.78 (0.64) *p* < .001
*z-score*,* p-value*	z = -0.07, *p* = .94		z = -0.46, *p* = .64	
***Roland Morris Disability Questionnaire***				
	1.30 (0.43) *p* = .002	1.29 (0.44) *p* = .003	-1.03 (0.38) *p* = .007	-1.28 (0.54) *p* = .017
*z-score*,* p-value*	z = 0.02, *p* = .98		z = 0.37, *p* = .71	
***Overall health rating***				
	-0.14 (0.10) *p* = .169	-0.14 (0.10) *p* = .163	0.08 (0.03) *p* = .009	0.11 (0.08) *p* = .127
*z-score*,* p-value*	z = -0.01, *p* = .99		z = -0.37, *p* = .71	

Standard errors are in parentheses

#### Pain interference

Average change (slopes) for PROMIS-16 and PROMIS-29 + 2 significantly predicted the average change for the ODI and RMDQ but not the overall health rating, with only trivial differences in estimates between the PROMIS-16 and PROMIS-29 + 2. In the parentheses, we first present the coefficient for PROMIS-16 (subscript P16), followed by the coefficient for the PROMIS-29 + 2 (subscript P29 + 2). On average, decreased pain interference predicted average decreases in the ODI (b_P16_=2.694, b_P29+2_=2.632) and RMDQ (b_P16_=1.304; b_P29+2_=1.290) over the three assessment waves.

#### Physical function

The average change for PROMIS-16 and PROMIS-29 + 2 predicted average change for the ODI, RMDQ, and overall health rating. In the parentheses, we first present the coefficient for PROMIS-16 followed by the coefficient for PROMIS-29 + 2. On average, decreases in physical function predicted larger increases in the ODI (b_P16_= -2.389; b_P29+2_= -2.777) and RMDQ (b_P16_= -1.030; b_P29+2_= -1.276) over the three assessment waves. Moreover, on average, improvements in physical function predicted larger increases in ratings of overall health with the PROMIS-16 (b = 0.084) but not significantly with the PROMIS-29 + 2 (b = 0.114), despite significantly comparable coefficient magnitudes (*p* = .71).

## Discussion

This paper expands prior work by longitudinally validating the eight PROMIS-16 domains across three waves of data by comparing performance to the PROMIS-29 + 2 in a sample of individuals with chronic low back pain. Across all eight domains, the PROMIS-16 captured comparable average baseline scores and changes over time. Additionally, random intercept and slope scores for individuals were highly correlated between the PROMIS-16 and PROMIS-29 + 2. Further, mean changes in physical function and pain interference represented as slopes comparably predicted mean change in ODI scores, RMDQ scores, and overall health ratings. Preliminary between-level results support the longitudinal validity and utility of the ultra-short PROMIS-16 profile domain scores for use in clinical settings and research.

Average baseline scores (i.e., intercepts) for the corresponding PROMIS-16 and PROMIS 29 + 2 domains were significantly different, but the effect sizes were minimal. Given the relatively large sample size, this was likely due to being highly powered to detect even trivial differences. Further, there was variability in the magnitude of the discrepancies. For instance, much smaller differences were observed for depression, anxiety, and pain interference than for physical function, fatigue, and social roles. That said, given that PROMIS domain scores are on a T distribution (Mean = 50, SD = 10), the largest average baseline difference was 0.13 SDs – an arguably ignorable difference. On the other hand, mean change over time was always statistically comparable between the PROMIS-16 and PROMIS 29 + 2, indicating the two-item PROMIS-16 domain scores were sensitive enough to produce comparable average change estimates over the 6 months.

This is promising for clinical and research settings. Short forms are appropriate and desirable in research settings if a reliable and valid discrete single domain is measured [32]. Prior psychometric work has demonstrated the reliability of PROMIS-16 domain scores (eight discrete domains) [9], and this study builds on this work by providing evidence supporting the longitudinal and predictive validity of the PROMIS-16. Additionally, short forms have a place in clinical screening, in which time burden, cost, and resources are major considerations [30]. The PROMIS-16 is a psychometrically sound fixed-length short profile measure that limits patient or respondent burden and the associated data collection costs while producing domain score change estimates comparable to longer, more time-intensive measures.

It is important to note that while average change score estimates were statistically comparable between the PROMIS-16 and PROMIS-29 + 2, this was observed with three time points over 6 months. Research is needed to evaluate and compare average change over a longer period and consider possible non-linear change. Moreover, this work was primarily focused on between-level effects. Future work should examine more nuanced within-person change, perhaps among a clinical sample where more change and individual variation are expected. Additionally, there was limited variability in overall health ratings over time, perhaps due to using a 5-point scale and that scores tended to be relatively stable over time. This may have contributed to the lack of prediction between slopes for PROMIS domains and overall health rating. While results were similar between the PROMIS-16 and PROMIS-29 + 2, longer forms will have greater precision, particularly in the extremes of the distribution. Further, this study used a sample of respondents with chronic lower back pain (CLBP) from an online nonprobability convenience sample. Future work is needed to evaluate the predictive validity of the PROMIS-16 and PROMIS-29 + 2 in different samples, including the general population and those with health conditions other than CLBP. It is also worth noting that while this sample included respondents who reported chronic low back pain, they were not necessarily receiving treatment or exposed to an event where pain may be affected. This may explain why we did not see mean changes over time in several domains. As such, future work should consider evaluating longitudinal validity in a clinical sample receiving treatment to determine if changes in other domains are comparable between the PROMIS-16 and PROMIS-29.

## Electronic supplementary material

Below is the link to the electronic supplementary material.

Supplementary Material 1
