# Supplementary material for: Longitudinal validation of the PROMIS-16 in a sample of adults in the United States with back pain
Source: Qual Life Res. 2024 Nov 6;34(1):35–42. doi: 10.1007/s11136-024-03826-6 (PMC11802292; doi:10.1007/s11136-024-03826-6)

Table S1. Means and Standard Deviations for All PROMIS-16 (P16) and PROMIS-29+2 (P29+2) Domains and Outcome Measures Across Timepoints

<table>
<tr><th></th><th>P29+2
M(SD)</th><th>P16
M(SD)</th></tr>
<tr><td>Anxiety Baseline</td><td>55.89 (9.55)</td><td>55.98 (9.76)</td></tr>
<tr><td>Anxiety 3 months</td><td>56.33 (9.78)</td><td>56.70 (9.73)</td></tr>
<tr><td>Anxiety 6 months</td><td>55.39 (9.81)</td><td>55.49 (9.90)</td></tr>
<tr><td>Cognitive Function Baseline</td><td>49.53 (7.75)</td><td>48.82 (8.17)</td></tr>
<tr><td>Cognitive Function 3 months</td><td>49.65 (7.89)</td><td>48.88 (8.18)</td></tr>
<tr><td>Cognitive Function 6 months</td><td>50.09 (7.87)</td><td>49.56 (8.36)</td></tr>
<tr><td>Depression Baseline</td><td>54.86 (10.03)</td><td>55.02 (9.91)</td></tr>
<tr><td>Depression 3 months</td><td>55.09 (10.29)</td><td>55.12 (10.24)</td></tr>
<tr><td>Depression 6 months</td><td>54.12 (10.28)</td><td>54.29 (10.16)</td></tr>
<tr><td>Fatigue Baseline</td><td>53.49 (9.94)</td><td>52.52 (9.56)</td></tr>
<tr><td>Fatigue 3 months</td><td>54.08 (9.78)</td><td>52.97 (9.30)</td></tr>
<tr><td>Fatigue 6 months</td><td>53.60 (10.07)</td><td>52.66 (9.57)</td></tr>
<tr><td>Physical Function Baseline</td><td>47.19 (8.65)</td><td>48.34 (8.14)</td></tr>
<tr><td>Physical Function 3 months</td><td>46.05(8.43)</td><td>47.58 (8.18)</td></tr>
<tr><td>Physical Function 6 months</td><td>46.33 (8.64)</td><td>47.56 (8.31)</td></tr>
<tr><td>Pain Interference Baseline</td><td>55.04 (8.22)</td><td>55.30 (8.00)</td></tr>
<tr><td>Pain Interference 3 months</td><td>54.59 (8.58)</td><td>54.81 (8.26)</td></tr>
<tr><td>Pain Interference 6 months</td><td>54.25 (8.70)</td><td>54.48 (8.41)</td></tr>
<tr><td>Sleep Disturbance Baseline</td><td>53.17 (9.04)</td><td>52.21 (8.32)</td></tr>
<tr><td>Sleep Disturbance 3 months</td><td>53.29 (9.06)</td><td>52.48 (8.32)</td></tr>
<tr><td>Sleep Disturbance 6 months</td><td>53.49 (9.15)</td><td>52.44 (8.33)</td></tr>
<tr><td>Social Roles Baseline</td><td>51.02 (9.21)</td><td>50.04 (9.08)</td></tr>
<tr><td>Social Roles 3 months</td><td>50.91 (9.35)</td><td>50.11 (9.08)</td></tr>
<tr><td>Social Roles 6 months</td><td>51.30 (9.39)</td><td>50.52 (9.07)</td></tr>
<tr><td>Overall health rating Baseline</td><td>3.15 (0.98)</td><td></td></tr>
<tr><td>Overall health rating 3 months</td><td>3.03 (0.97)</td><td></td></tr>
<tr><td>Overall health rating 6 months</td><td>3.15 (0.96)</td><td></td></tr>
<tr><td>Oswestry Disability Index Baseline</td><td colspan="2">22.62 (16.12)</td></tr>
<tr><td>Oswestry Disability Index 3 months</td><td colspan="2">21.65 (17.21)</td></tr>
<tr><td>Oswestry Disability Index 6 months</td><td colspan="2">20.79 (17.00)</td></tr>
<tr><td>Roland Morris Disability Questionnaire Baseline</td><td colspan="2">8.22 (6.49)</td></tr>
<tr><td>Roland Morris Disability Questionnaire 3 months</td><td colspan="2">7.15 (6.79)</td></tr>
<tr><td>Roland Morris Disability Questionnaire 6 months</td><td colspan="2">6.79 (6.63)</td></tr>
</table>

Supplemental Spaghetti Plots of Individual Trajectories by Domain with P29+2 First Followed by P16

Anxiety

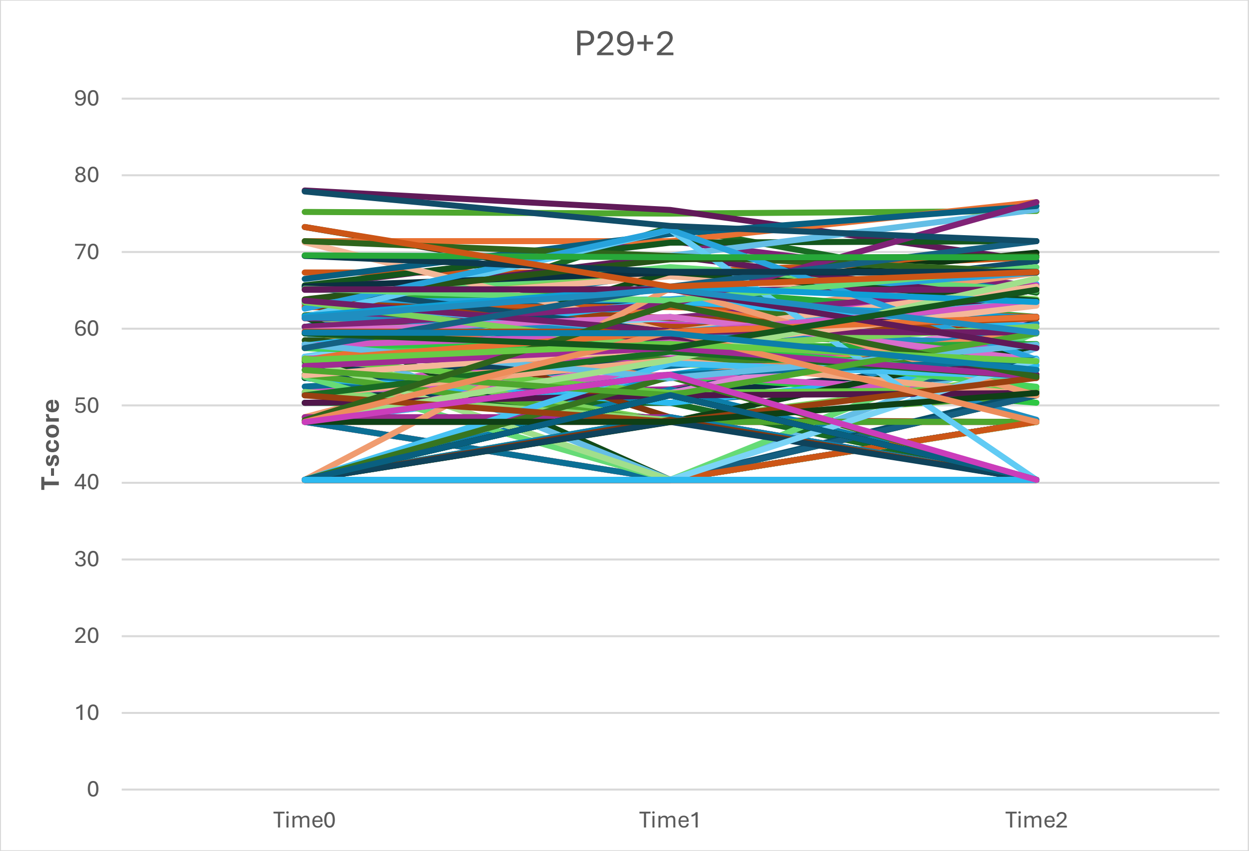

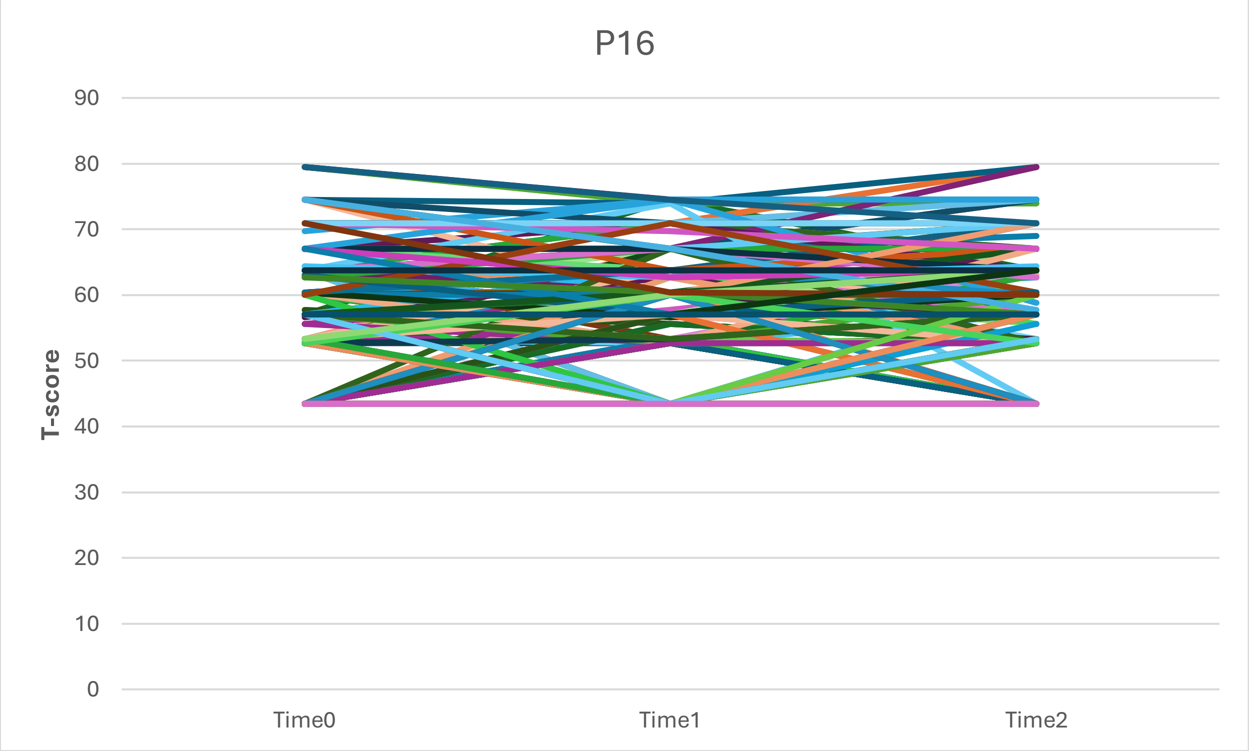

Cognitive Function

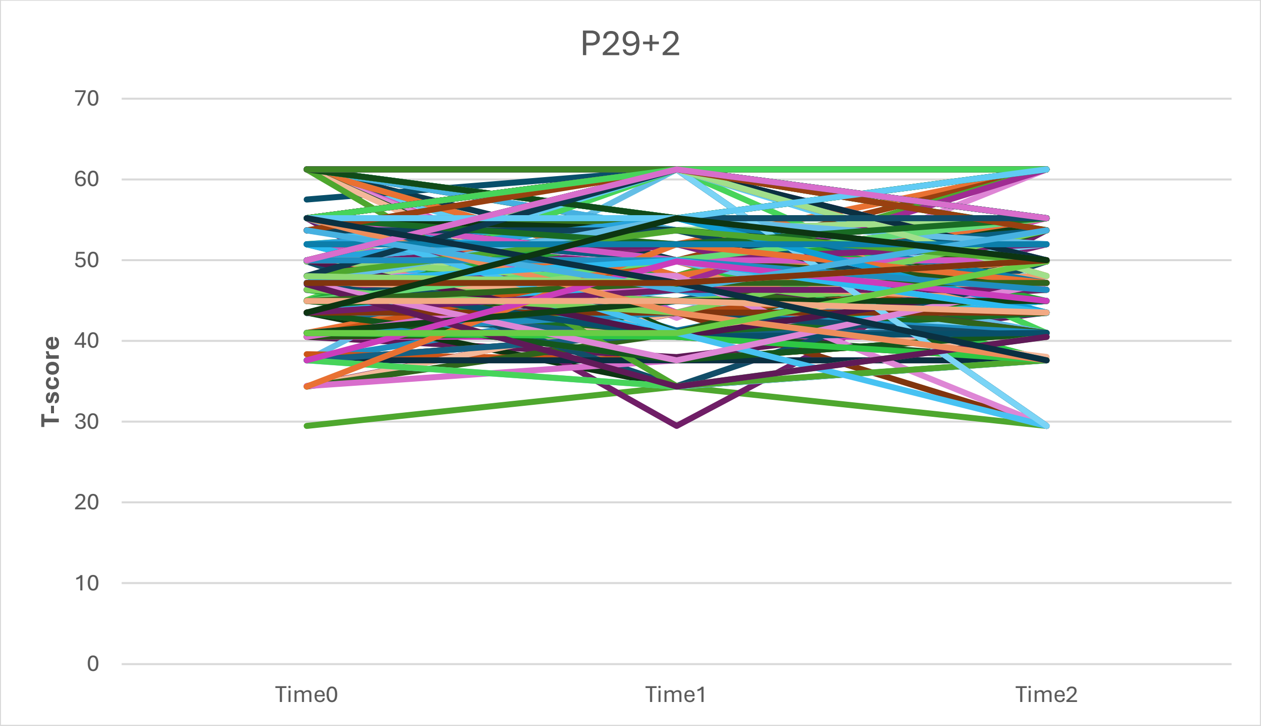

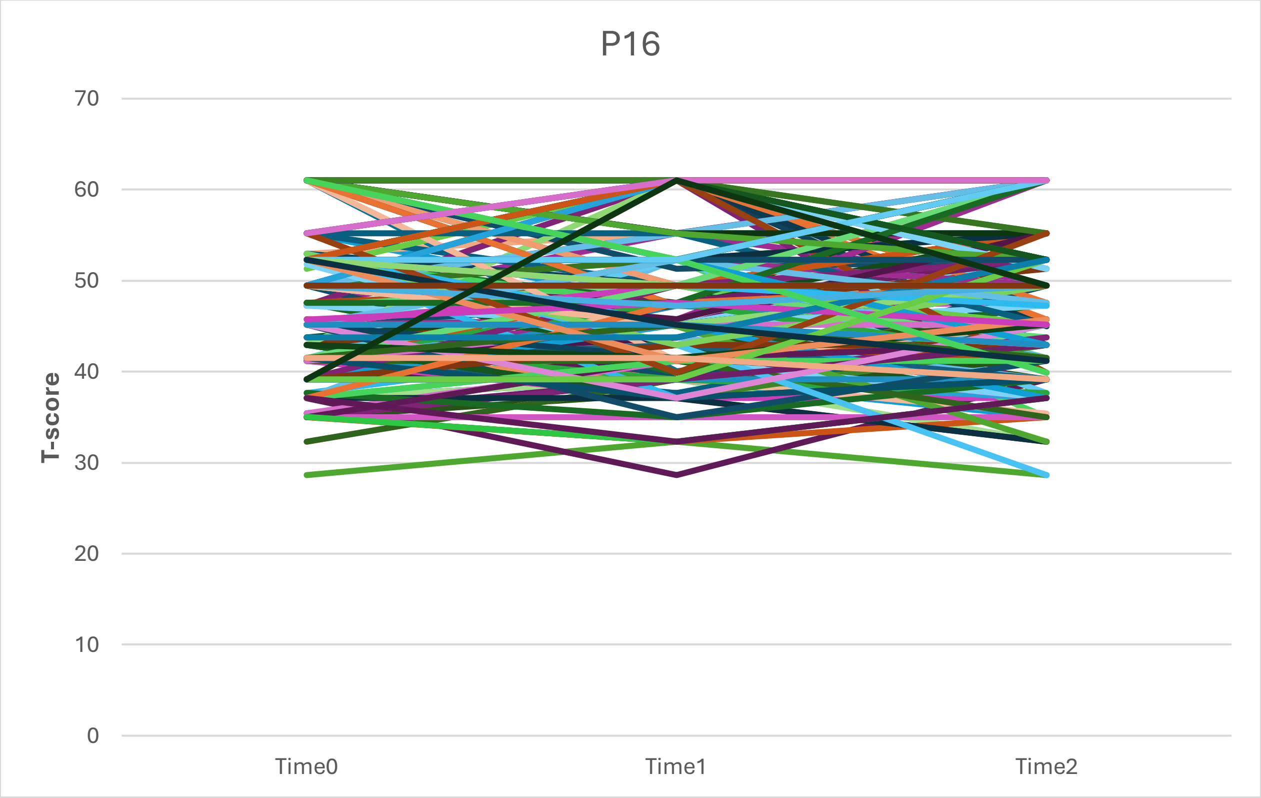

Depression

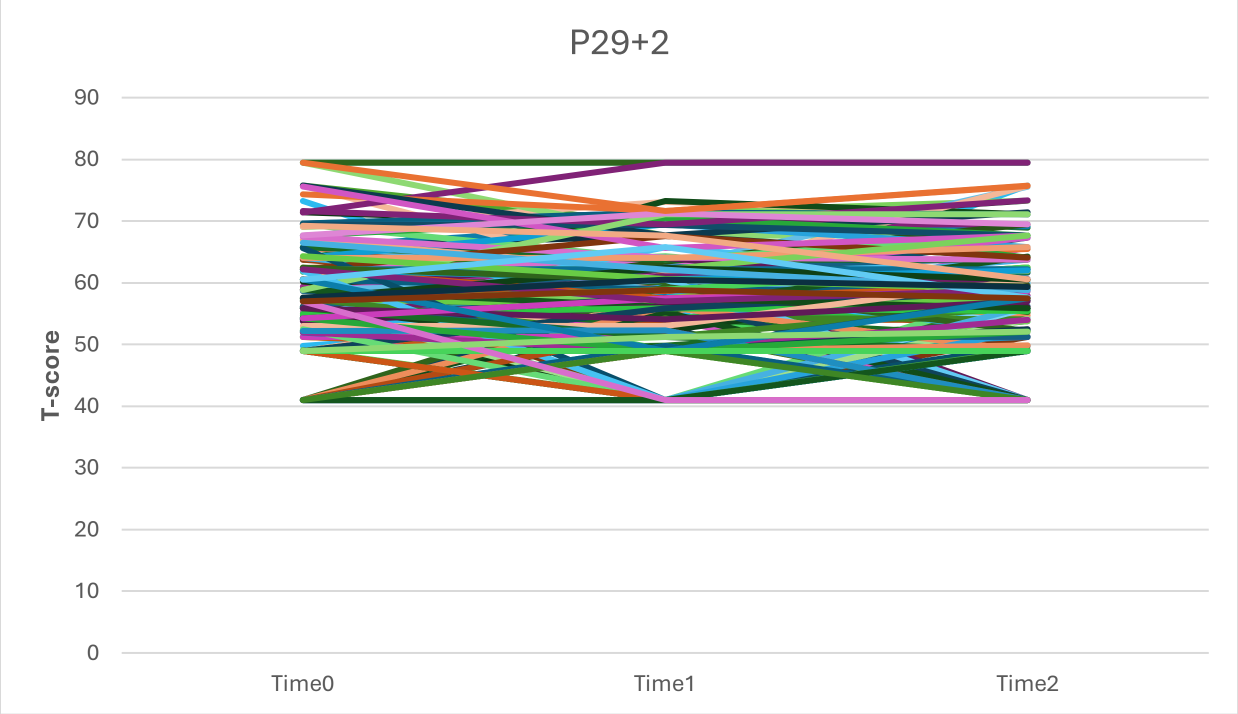
P29+2
90
80
70
60
50
40
30
20
10
0
T-score
Time0
Time1
Time2

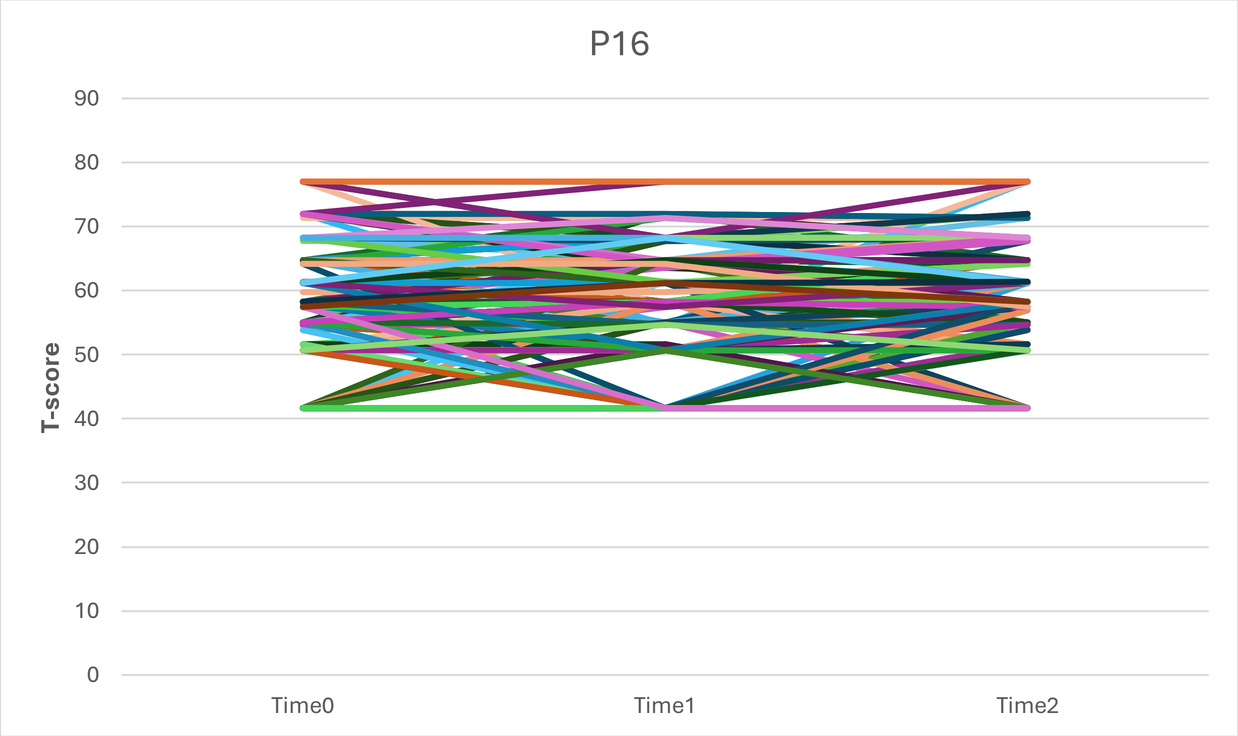
P16
90
80
70
60
50
40
30
20
10
0
T-score
Time0
Time1
Time2

Fatigue

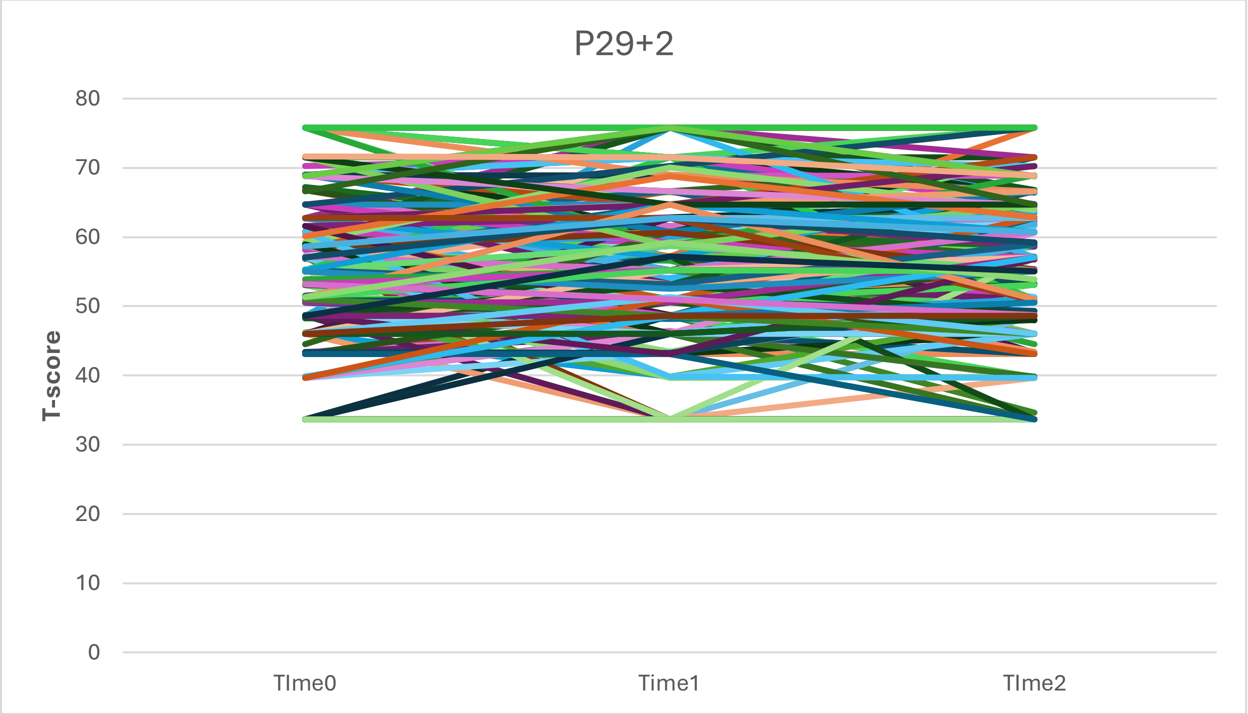
P29+2
T-score
80
70
60
50
40
30
20
10
0
TIme0
Time1
TIme2

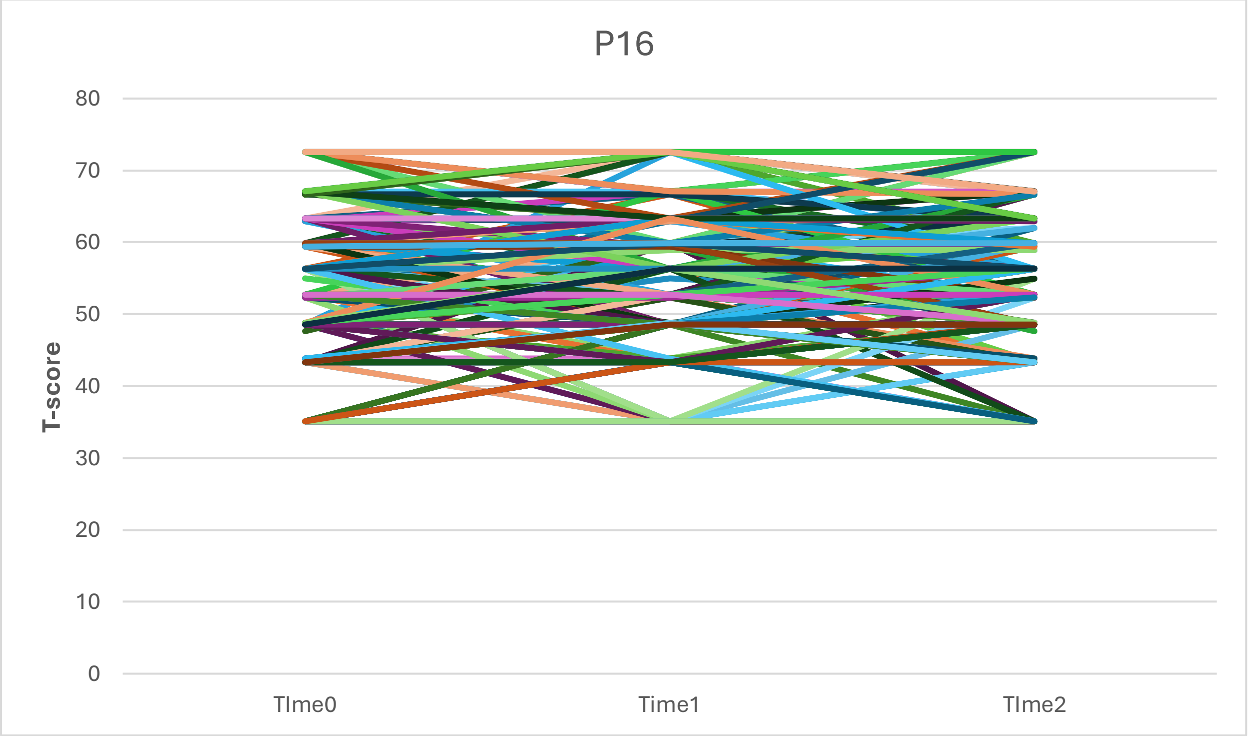
P16
T-score
80
70
60
50
40
30
20
10
0
TIme0
Time1
TIme2

Physical Function

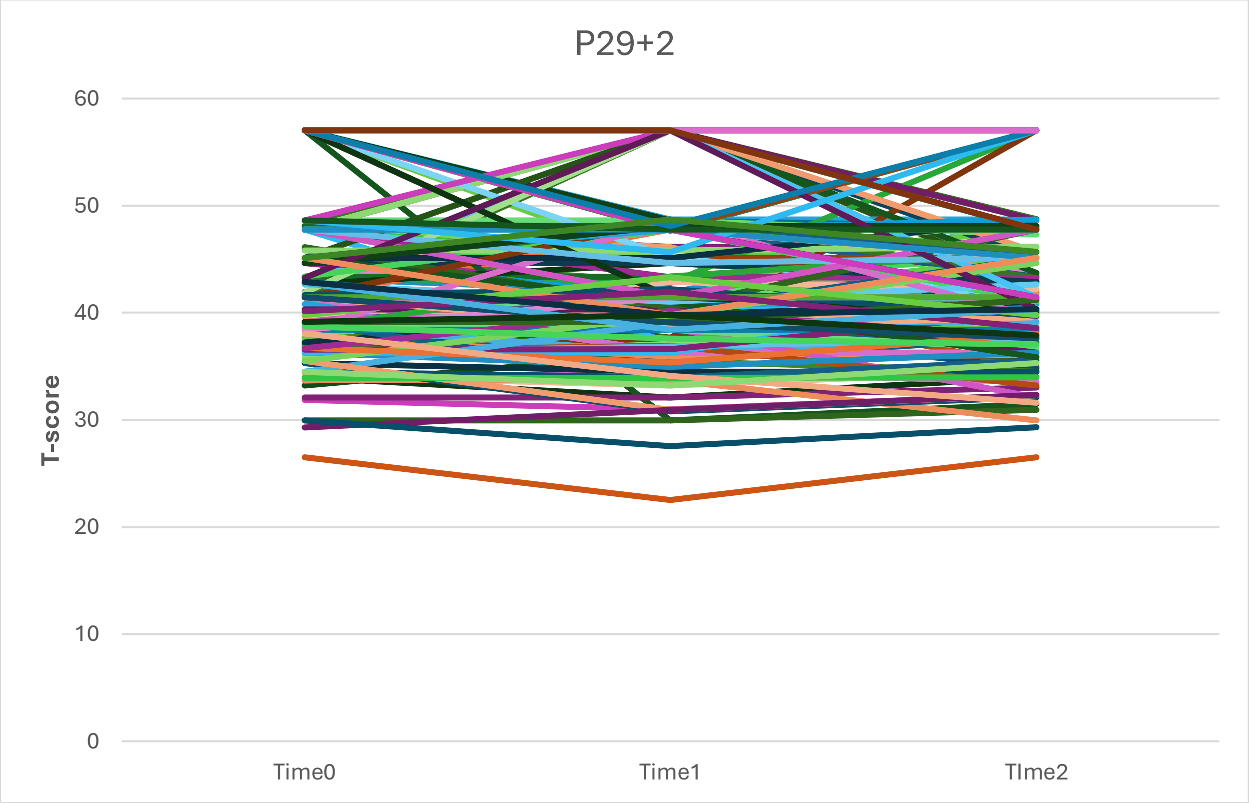

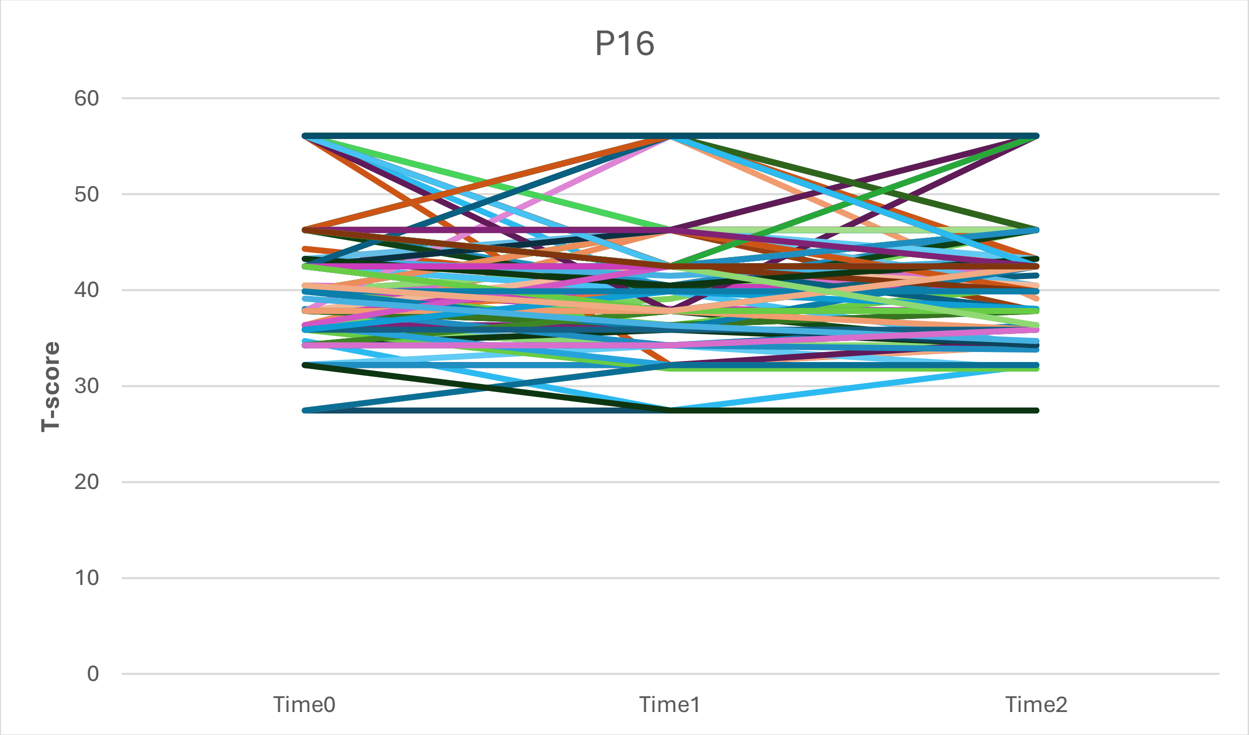

Pain interference

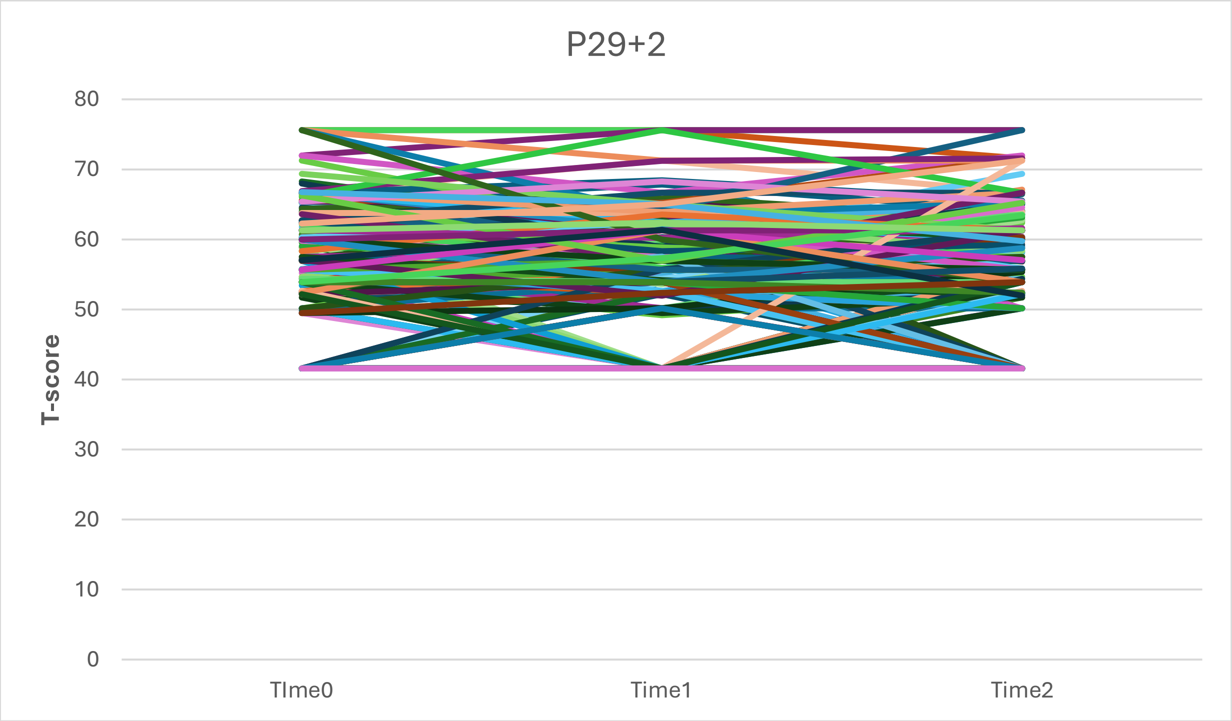

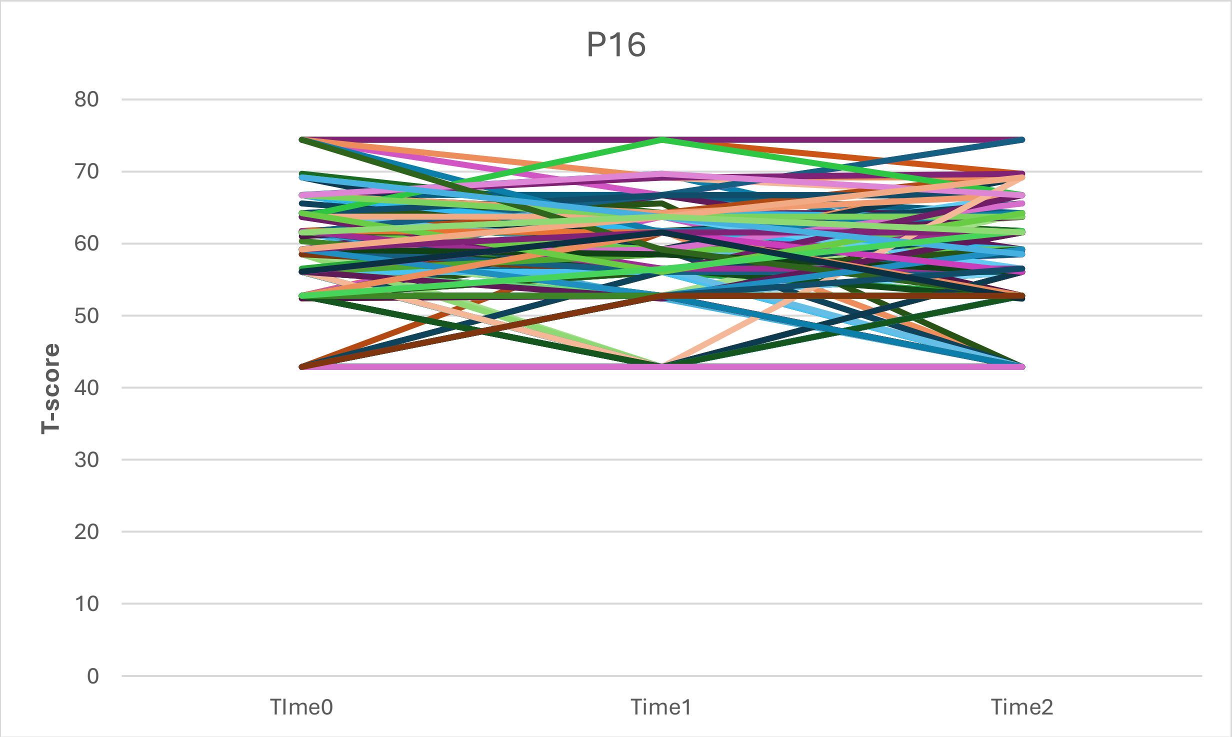

Sleep Disturbances

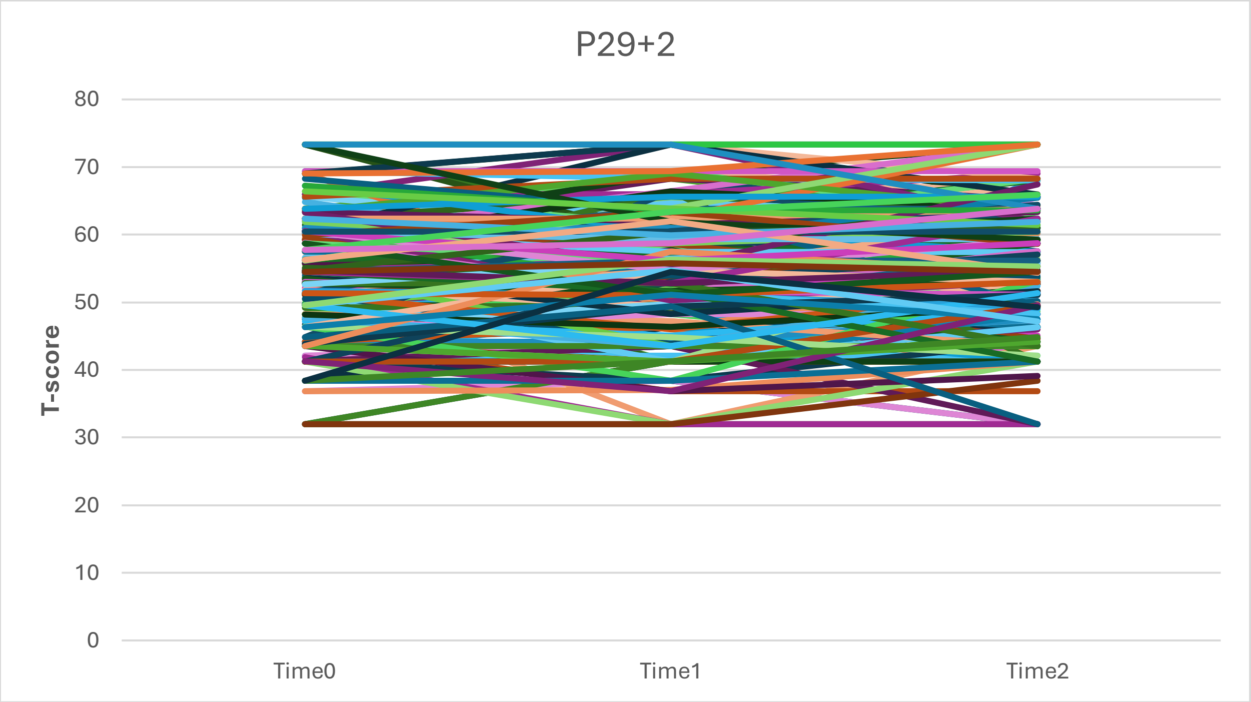

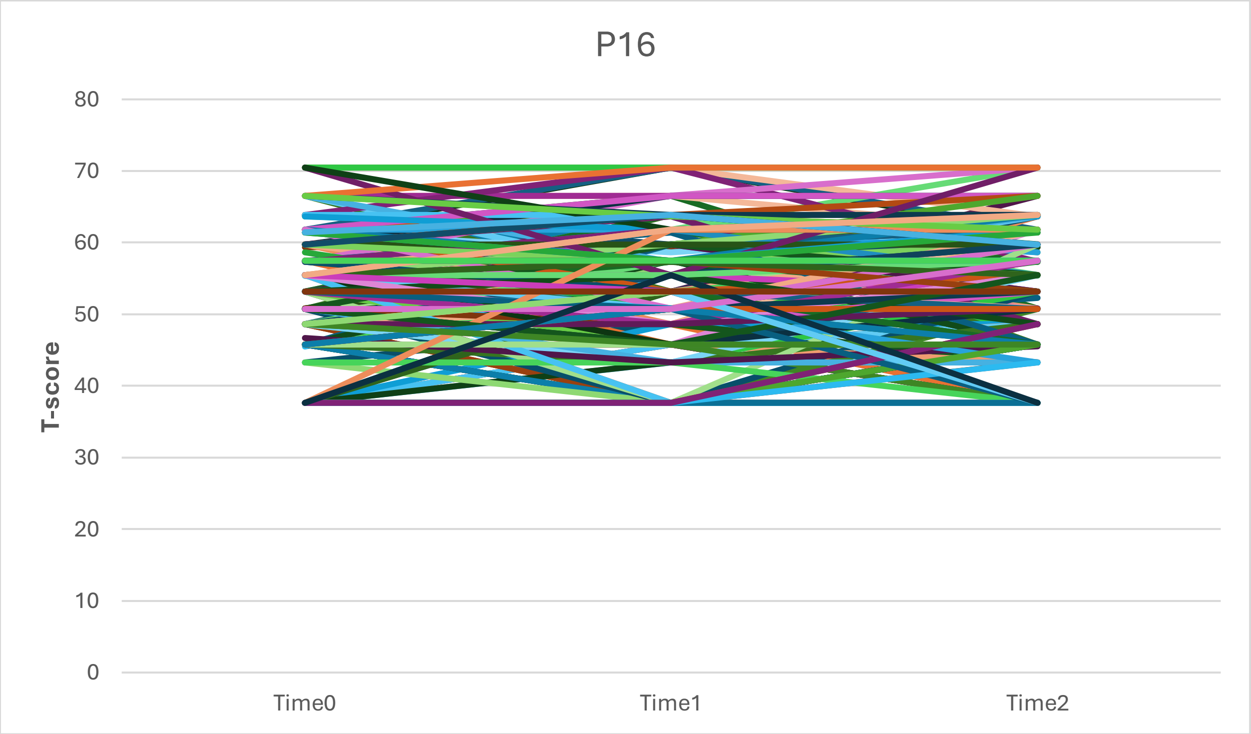

Social Roles

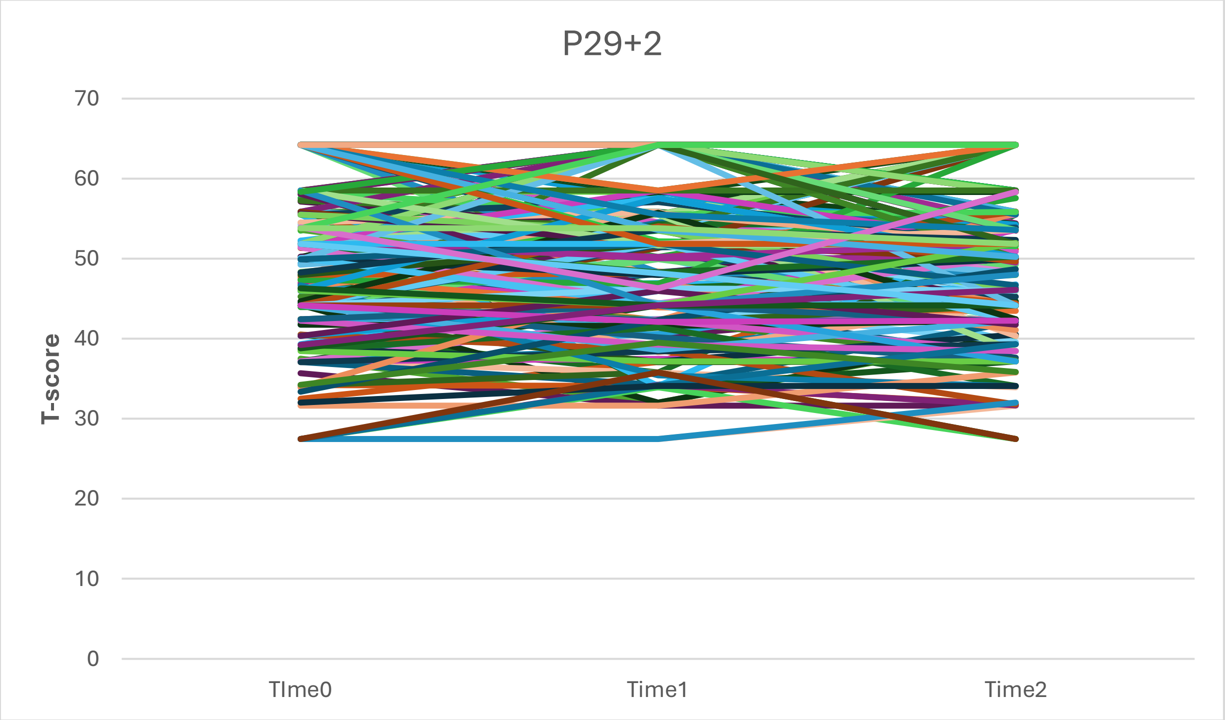

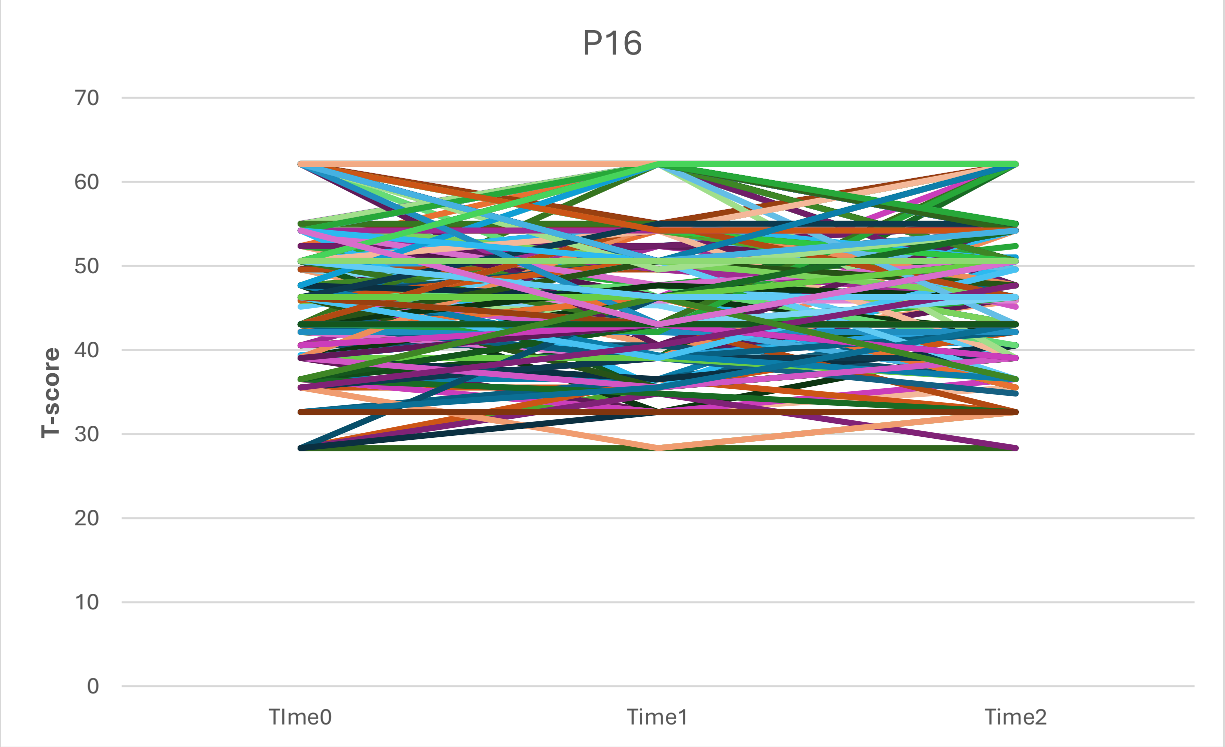

Oswestry Disability Index (ODI)

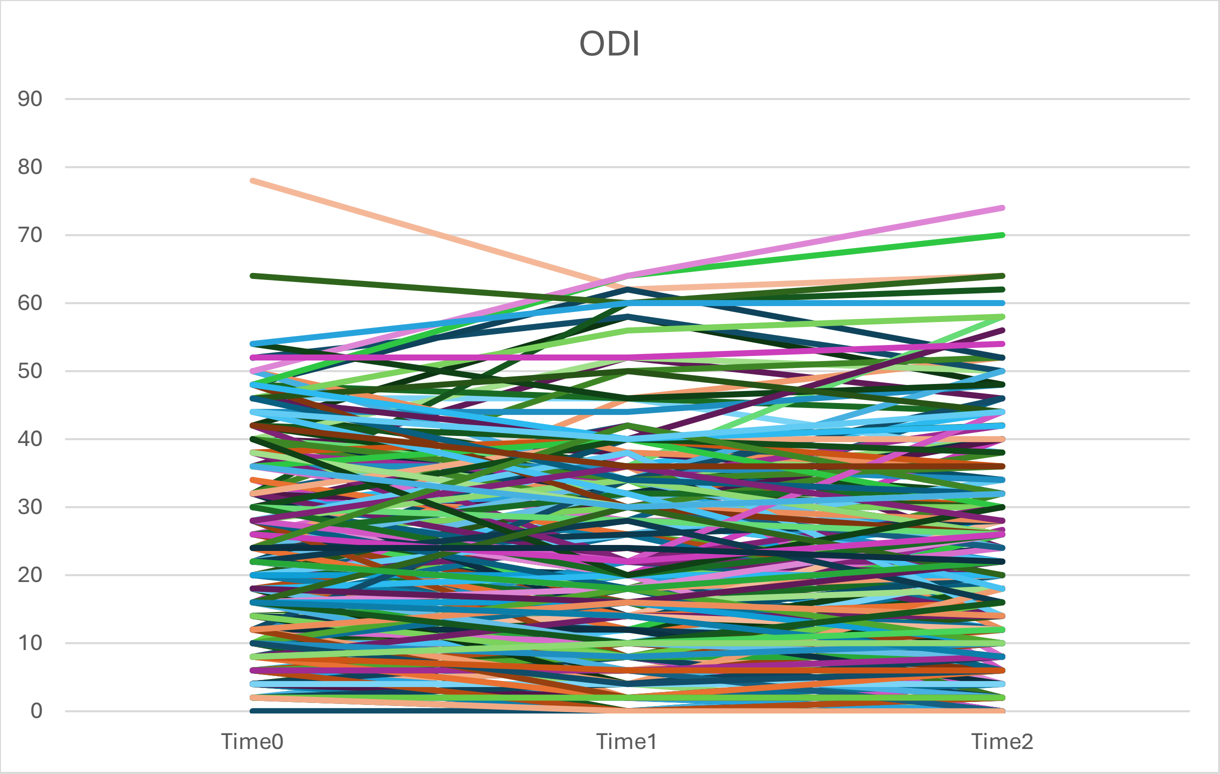

Roland Morris Disability Questionnaire (RMDQ)

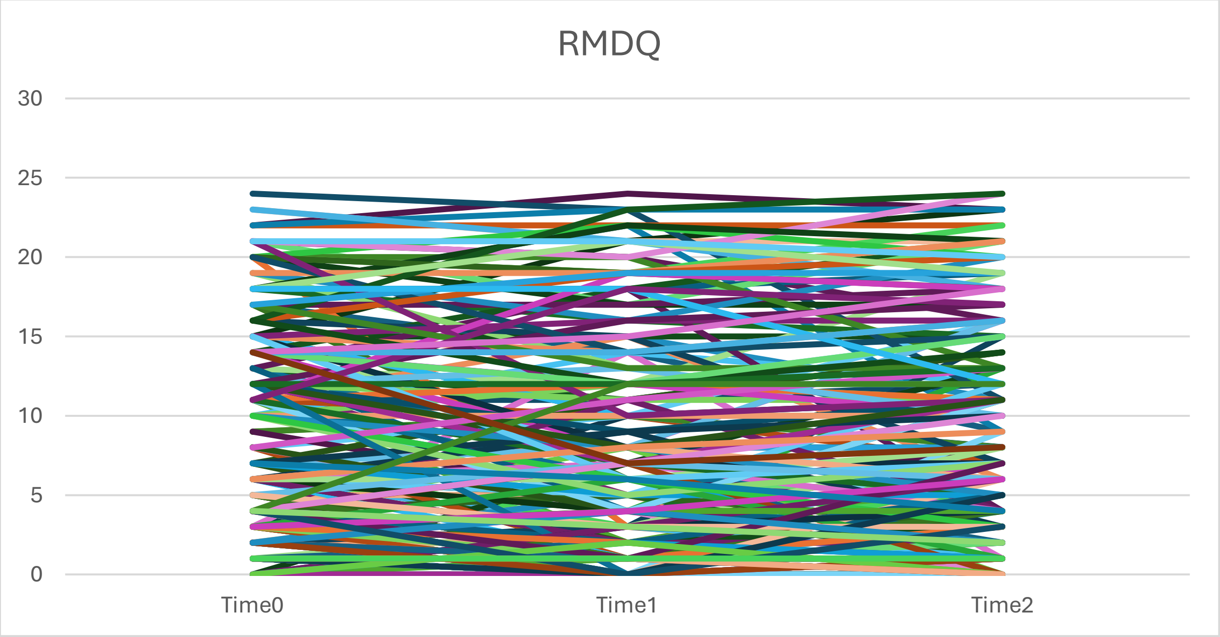

Overall health rating

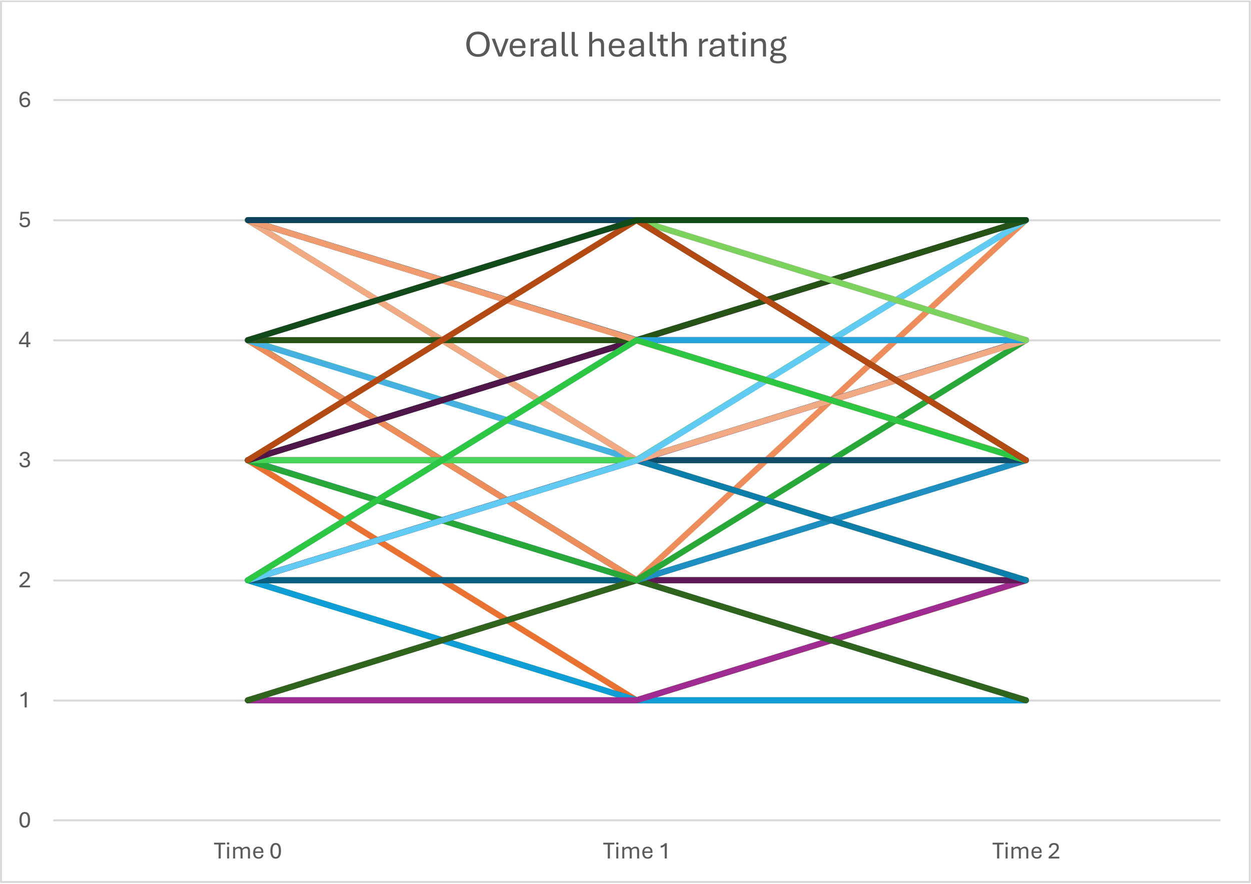

Supplement: Supplementary file 1 — Supplementary Material 1 [file 11136_2024_3826_MOESM1_ESM.pdf]
